# In Silico Targeting and Immunological Profiling of PpiA in *Mycobacterium tuberculosis*: A Computational Approach

**DOI:** 10.3390/pathogens14040370

**Published:** 2025-04-09

**Authors:** Mohammad J. Nasiri, Lily Rogowski, Vishwanath Venketaraman

**Affiliations:** 1 Department of Microbiology, School of Medicine, Shahid Beheshti University of Medical Sciences, Tehran 19839-69411, Iran; mj.nasiri@hotmail.com; 2 College of Osteopathic Medicine of the Pacific, Western University of Health Sciences, Pomona, CA 91766-1854, USA; lily.rogowski@westernu.edu

**Keywords:** tuberculosis vaccine, peptidyl-prolyl isomerase A (PpiA), immunoinformatic, epitope prediction, B-cell epitopes, T-cell epitopes, antigenicity

## Abstract

Tuberculosis (TB) remains a leading cause of mortality, with drug resistance highlighting the need for new vaccine targets. Peptidyl-prolyl isomerase A (PpiA), a conserved Mycobacterium tuberculosis (Mtb) protein, plays a role in bacterial stress adaptation and immune evasion, making it a potential target for immunotherapy. This study uses computational methods to assess PpiA’s antigenicity, structural integrity, and immunogenic potential. The PpiA sequence was retrieved from NCBI and analyzed for antigenicity and allergenicity using VaxiJen, AllerTOP, and AllergenFP. Physicochemical properties were evaluated using ProtParam, and structural models were generated through PSIPRED and SWISS-MODEL. Structural validation was performed with MolProbity, QMEANDisCo, and ProSA-Web. B-cell epitopes were predicted using BepiPred 2.0 and IEDB, while T-cell epitopes were mapped via IEDB’s MHC-I and MHC-II tools. Epitope conservation across Mtb strains was confirmed using ConSurf. Results indicate PpiA is highly antigenic, non-allergenic, and stable, with several immunogenic epitopes identified for both B- and T-cells. This study supports PpiA as a promising immunogenic target for TB vaccine development.

## 1. Introduction

Tuberculosis (TB) remains a leading cause of global morbidity and mortality, with 10 million cases and 1.25 million deaths in 2023 [1]. Despite the BCG vaccine and antibiotics, the disease burden persists due to limited intervention efficacy and the rise of multidrug-resistant (MDR) *Mycobacterium tuberculosis* (Mtb), underscoring the need for novel therapeutic strategies [2,3,4,5,6].

A major challenge in TB control is Mtb’s ability to evade the immune system and persist in a latent state [7]. Mtb employs various virulence factors, including the iron-regulated peptidyl-prolyl isomerase PpiA (Rv1508c), which is crucial for adaptation and survival under stress [8,9,10,11]. PpiA catalyzes the cis–trans isomerization of proline residues, aiding protein folding during iron limitation, a condition common in Mtb infections, highlighting its role in bacterial persistence and virulence [8,9,10,11].

This protein is highly conserved across Mtb strains, making it an attractive target for immune-based therapies and diagnostic approaches. Furthermore, PpiA has been identified as a promising candidate for immunological studies due to its potential to induce both cellular and humoral immune responses in infected individuals. PpiA is a secreted, cell wall-associated protein, making it accessible to the host immune system [8,9,10,11]. Its surface exposure and immunogenicity suggest it could be a promising target for immunological interventions. While many studies focus on new TB vaccines and improving BCG efficacy, PpiA remains underexplored as a target for vaccine or antibody therapies, presenting an opportunity for computational approaches to design novel immunotherapeutics [8,9,10,11]. Targeting PpiA could not only contribute to a better understanding of Mtb’s molecular mechanisms but also open avenues for developing more effective diagnostic and therapeutic strategies against tuberculosis.

Immunoinformatics, which uses computational tools to predict antigenic epitopes, plays a crucial role in the design of vaccines, enabling the identification of potential targets without the need for time-consuming experimental validation. This approach has already been successfully applied to other Mtb proteins, such as ESAT-6 and CFP-10, which have been studied for their immunogenic potential and use in diagnostics and vaccines [12]. By analyzing the MHC-I and MHC-II binding properties of various Mtb antigens, researchers can identify peptide epitopes that are likely to elicit robust immune responses, thus advancing vaccine development.

In addition to computational approaches, several strategies have been employed in the development of vaccines against Mtb, such as the enhancement of the BCG vaccine, subunit vaccines, DNA vaccines, and viral-vectored vaccines [13,14,15]. However, the development of an effective vaccine remains a challenge, particularly against latent and drug-resistant strains of Mtb, which persist in the host’s immune system. The immune response to Mtb infection involves both cellular and humoral immunity, with CD4+ T-cells playing a pivotal role in controlling the infection [16]. The identification of MHC-I and MHC-II binding epitopes is crucial in designing vaccines that can stimulate both arms of the immune system effectively, offering the possibility of improving the host’s immune response against Mtb.

In this study, we use advanced computational approaches to identify and characterize PpiA as a novel immunological target for vaccine development and immunotherapy. By analyzing its structural and functional properties, we aim to uncover key epitopes and binding sites that could enable the design of targeted interventions. This approach provides a basis for developing new therapeutic strategies to combat TB, with a focus on enhancing immune responses against latent and drug-resistant strains of Mtb.

## 2. Materials and Methods

### 2.1. Protein Sequence Retrieval and Analysis

The nucleotide sequence of PpiA (Rv1508c) from Mtb was retrieved in FASTA format from the National Center for Biotechnology Information (NCBI) database (http://www.ncbi.nlm.nih.gov) [17]. The corresponding amino acid sequence of PpiA was obtained from the NCBI protein database (https://www.ncbi.nlm.nih.gov/protein/) [17] (All databases were accessed on 1 January 2025). The amino acid sequence of PpiA from Mtb H37Rv was subjected to a blastp search against the non-redundant protein sequence (nr) database in the NCBI server (http://blast.ncbi.nlm.nih.gov/Blast.cgi). Sequences with >90% identity, >90% coverage, and E-value < 10^−4^ were selected [17].

### 2.2. Prediction of Antigenicity, Allergenicity, and Physicochemical Properties

To evaluate the potential of PpiA (Rv1508c) as an immunological target, we employed computational tools to predict its antigenicity, allergenicity, and physicochemical properties.

#### 2.2.1. Antigenicity Prediction

The antigenic potential of PpiA was assessed using VaxiJen v2.0 (http://www.ddg-pharmfac.net/vaxijen/VaxiJen/VaxiJen.html) [17,18]. The protein sequence was entered in FASTA format, and predictions were made using the “Bacteria” model. A threshold value of 0.4 was set to classify the protein as either an antigen or non-antigen.

#### 2.2.2. Allergenicity Prediction

The allergenicity of PpiA was predicted using three different tools: AllerTOP v2.0 (https://www.ddg-pharmfac.net/AllerTOP/), AllergenFP v1.0 (http://ddg-pharmfac.net/AllergenFP/), and AlgPred (https://webs.iiitd.edu.in/raghava/algpred/) [17,19]. Each tool was used to analyze the protein sequence and predict its potential to cause allergic reactions.

#### 2.2.3. Physicochemical Properties

The physicochemical properties of PpiA, including molecular weight, theoretical isoelectric point (pI), instability index, aliphatic index, and grand average of hydropathicity (GRAVY), were determined using the ProtParam tool on the ExPASy server (https://web.expasy.org/protparam/) [17,20,21]. These properties provide important information regarding the protein’s stability, solubility, and overall structural characteristics.

### 2.3. Secondary and Tertiary Structure Prediction

To understand the structural features of PpiA, we predicted its secondary and tertiary structures using advanced computational tools.

#### 2.3.1. Secondary Structure Prediction

The secondary structure of PpiA, including alpha-helices, beta-sheets, and coils, was predicted using PSIPRED v4.0 (http://bioinf.cs.ucl.ac.uk/psipred/) [17,22]. The protein sequence was submitted to the server, and the results were visualized to identify regions of structural stability and flexibility.

#### 2.3.2. Tertiary Structure Prediction

The 3D structure of PpiA was modeled using SWISS-MODEL (https://swissmodel.expasy.org/) [23,24]. The sequence was aligned with suitable templates from the Protein Data Bank (PDB), and the best model was selected based on the GMQE (Global Model Quality Estimation) and QMEAN scores [25]. Additionally, MolProbity was used to evaluate Ramachandran plot statistics, rotamer outliers, and clash scores [26,27,28].

#### 2.3.3. Model Validation

The models were validated using ProSA-Web (https://prosa.services.came.sbg.ac.at/prosa.php) to assess their overall quality and Z-scores [29].

### 2.4. B-Cell Epitope Mapping

B-cell epitope mapping was conducted using the Immune Epitope Database (IEDB) (https://www.iedb.org/) to identify potential protective epitopes for vaccine development [30,31,32,33]. The primary sequence of PpiA was submitted to IEDB’s B-cell epitope prediction tools. Linear epitopes were predicted using the BepiPred 2.0 algorithm, which combines a hidden Markov model and a propensity scale method to identify B-cell receptor-binding regions. Conformational (discontinuous) epitopes were predicted using IEDB’s tool based on protein structure or modeled structure.

IEDB’s tools evaluate multiple factors such as surface accessibility and physicochemical properties, and epitopes above the defined cut-off threshold were selected for further analysis. The identified epitopes were cross-referenced with the existing literature to ensure their relevance for eliciting a protective immune response against Mtb.

### 2.5. T-Cell Epitope Prediction

T-cell epitope prediction was carried out using the IEDB (https://www.iedb.org/) to identify potential peptides that could bind to major histocompatibility complex (MHC) molecules and trigger immune responses [31,33].

#### 2.5.1. MHC-I Epitope Prediction

For MHC-I epitopes, the IEDB MHC-I Binding Prediction tool was used to predict peptides that are likely to bind to various MHC-I alleles [31,33]. This tool utilizes an algorithm based on quantitative binding affinity data, which estimates how well a peptide will interact with different MHC-I molecules. Peptides with high binding affinity (IC50 < 50 nM) were selected for further analysis as they are more likely to elicit a cytotoxic T-cell response. The HLA-A*0201 allele, a common MHC class I molecule in the human population, was included in the predictions, as it is frequently used in epitope prediction studies and is known for its relevance in human immune responses.

#### 2.5.2. MHC-II Epitope Prediction

For MHC-II epitopes, the IEDB MHC-II Binding Prediction tool was employed to predict peptide-MHC-II interactions [31,33]. This tool predicts the binding of peptides to a range of MHC-II alleles, which is essential for the activation of helper T-cells. Peptides with high predicted binding affinities were chosen for further investigation, as they are expected to stimulate CD4+ T-cells and contribute to the overall immune response.

The resulting predicted T-cell epitopes for both MHC-I and MHC-II were validated by comparing them with available experimental data and the literature to ensure their relevance for eliciting both cytotoxic and helper T-cell responses in TB.

## 3. Results

### 3.1. Protein Sequence Retrieval and Analysis

**Figure 1:**
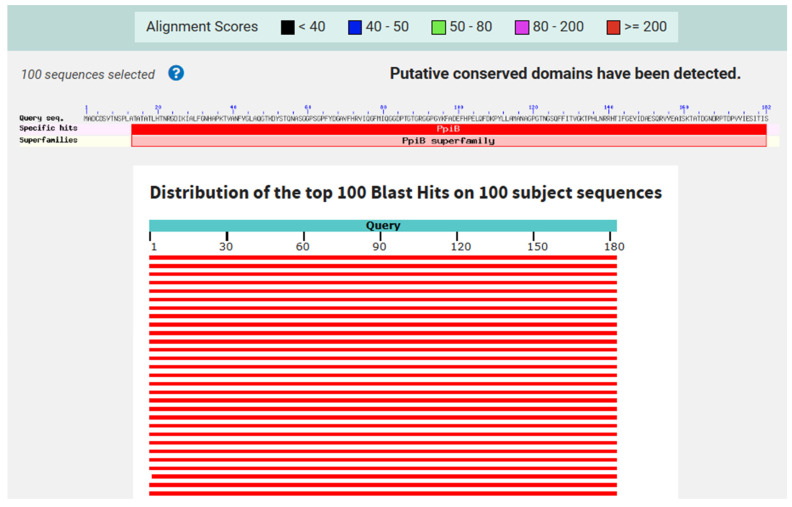
Sequence conservation of PpiA across various Mtb strains. The complete PpiA sequence was retrieved from Mtb H37Rv (NP_214523.1) and analyzed using BLASTp against multiple Mtb strains, including Mtb H37Rv, Mtb Beijing, Mtb India, Mtb L2, Mtb EAI, Mtb Haarlem, Mtb XDR, and Mtb KZN. The graphic summarizes the results of the multiple sequence alignment, highlighting that PpiA is highly conserved across these selected strains. Strains selected for analysis were based on geographical distribution and their relevance to clinical tuberculosis cases worldwide. The sequence conservation observed across these strains suggests that PpiA may serve as a potential target for broad-spectrum vaccine or diagnostic approaches.

We first performed a gene sequence alignment of the PpiA gene (Rv1508c) across various Mtb strains using the NCBI database. The alignment was conducted to assess the sequence variability and identify conserved regions in the gene. This initial gene sequence alignment allowed us to examine the degree of genetic conservation of PpiA across the strains, providing a foundation for further protein sequence analysis. Afterward, we retrieved the full protein sequence of PpiA (Rv1508c) from Mtb H37Rv (NP_214523.1) and conducted a BLAST 1.4.0 search of the complete PpiA sequence against multiple Mtb strains. The results revealed that the PpiA protein is highly conserved across these strains. The multiple sequence alignment of the full PpiA protein sequence showed that 180 out of 182 residues (98.9%) were conserved among the Mtb strains, indicating a high level of sequence conservation and suggesting its potential as a universal target for immunological interventions (Figure 1).

### 3.2. Antigenicity, Allergenicity, and Physicochemical Properties

The antigenicity prediction for PpiA, conducted using the VaxiJen tool, returned a score of 0.7191, which is above the threshold for bacterial antigens, classifying PpiA as a probable antigen. This high score indicates that PpiA has a strong likelihood of being recognized by the immune system.

The allergenicity prediction from AllerTOP, AllergenFP, and AlgPred indicated that PpiA is non-allergenic, suggesting that it is unlikely to induce allergic reactions, which enhances its suitability for therapeutic applications.

Regarding physicochemical properties, the PpiA protein consists of 182 amino acids, with a molecular weight of 19,239.40 Da and an isoelectric point (pI) of 5.81, which suggests that PpiA will be negatively charged at physiological pH. The amino acid composition is dominated by glycine (12.1%) and alanine (9.3%), contributing to flexibility and stability, respectively. The charge distribution includes 18 negatively charged residues (Asp + Glu) and 14 positively charged residues (Arg + Lys), supporting the protein’s overall negative charge. The instability index was calculated at 23.60, classifying PpiA as a stable protein. The aliphatic index of 67.58 suggests favorable thermal stability, while the grand average of hydropathicity (GRAVY) of −0.303 indicates that the protein is hydrophilic. Furthermore, the estimated half-life of PpiA is 30 h in mammalian reticulocytes (in vitro), greater than 20 h in yeast (in vivo), and more than 10 h in *E. coli* (in vivo), further confirming its stability across various biological systems.

### 3.3. Prediction of PpiA Tertiary and Secondary Structures

#### 3.3.1. Secondary Structure Prediction

**Figure 2:**
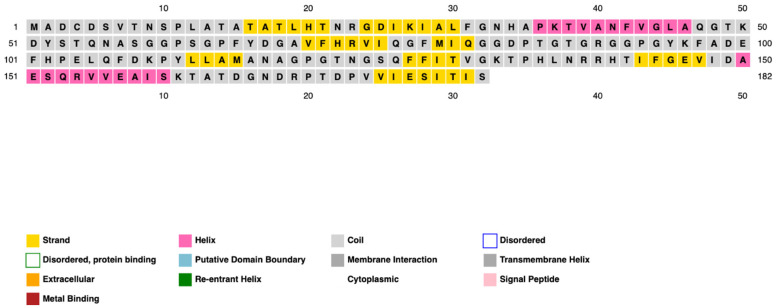
Predicted secondary structure of PpiA, illustrating the distribution of alpha-helices and beta-sheets within the protein. Alpha-helices are prominently present, contributing to the protein’s overall stability, while beta-sheets provide structural integrity. These features indicate potential regions of functional importance and possible sites for immune recognition. The secondary structure was predicted using PSIPRED.

**Figure 3:**
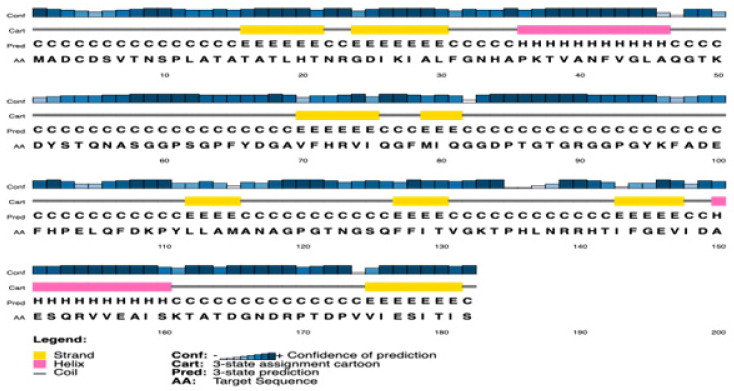
Visualization of PpiA’s secondary structure, highlighting exposed regions that may serve as targets for immune system recognition. The presence of alpha-helices and beta-sheets suggests a well-ordered structure critical for the protein’s stability and function. This visualization was created using SWISS-MODEL for structural modeling and PyMOL for structural visualization.

The secondary structure of PpiA was successfully predicted, revealing a significant presence of alpha-helices and beta-sheets. These structural elements are crucial for maintaining the protein’s stability and functionality. The predicted secondary structures highlight regions of PpiA that are likely to be exposed and could serve as potential targets for immune system recognition (Figure 2 and Figure 3).

#### 3.3.2. Tertiary Structure Prediction

**Figure 4:**
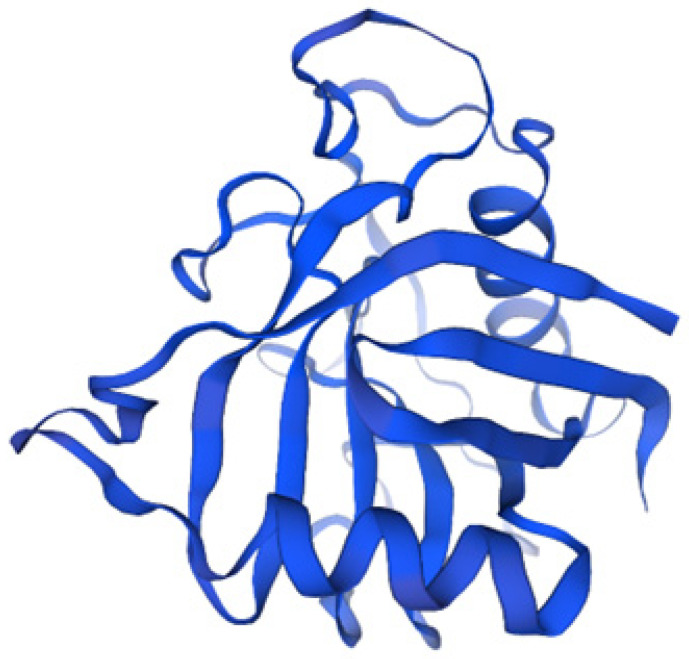
Predicted 3D structure of PpiA generated using SWISS-MODEL, based on the Mtb PpiA template (1w74.1.A). The model achieved a GMQE score of 0.93, indicating high reliability and structural resemblance to the target. This high-confidence model provides valuable insights into the spatial arrangement of PpiA, which is essential for understanding its stability, functionality, and potential interactions. Structural modeling was performed using SWISS-MODEL.

The SWISS-MODEL prediction produced a high-quality model with a GMQE score of 0.93, indicating a strong resemblance to the target structure. The model was built using the PpiA structure from Mtb (template 1w74.1.A), which provided a high level of confidence in the predicted 3D structure (Figure 4).

**Figure 5:**
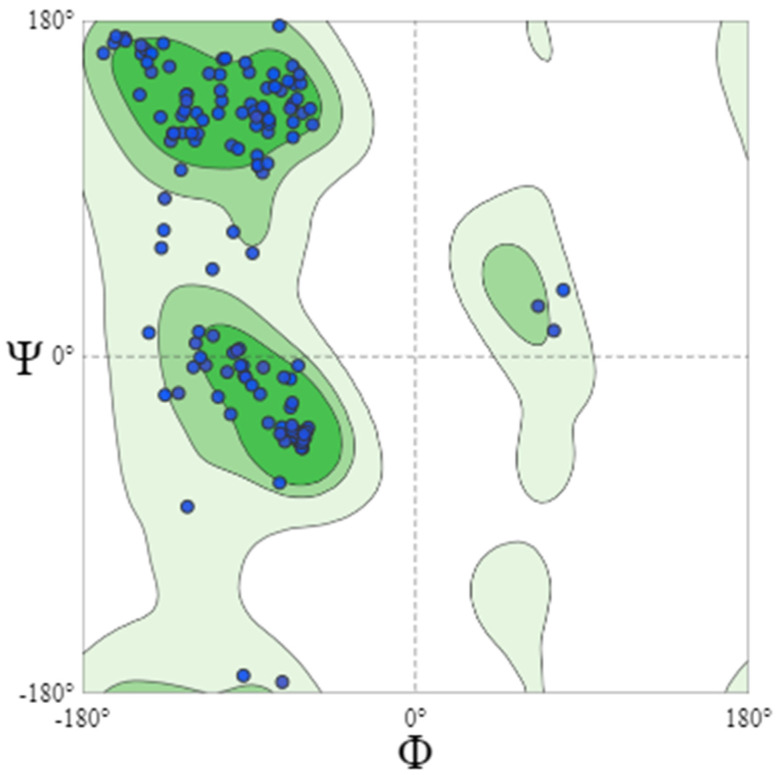
Ramachandran plot analysis of the predicted PpiA model, demonstrating the distribution of backbone dihedral angles (Φ, Ψ) for all residues. The majority of residues (93.49%) are located within the favored regions (dark green), indicating a well-folded and structurally reliable model. The minimal presence of outliers suggests a high-quality prediction with well-defined secondary structure elements, further supporting the model’s accuracy and stability. Ramachandran analysis was performed using MolProbity.

Further evaluation of the model through QMEANDisCo Global and MolProbity scores supported the overall quality of the structure. The QMEANDisCo Global score of 0.93 ± 0.07 suggests that the model’s quality is comparable to high-quality protein structures from the Protein Data Bank (PDB). The MolProbity score of 1.81 and the low clash score (0.79) indicate minimal steric clashes and a well-optimized structure. Additionally, 93.49% of the residues were found in the favorable regions of the Ramachandran plot, further supporting the model’s reliability (Figure 5).

#### 3.3.3. Model Validation

**Figure 6:**
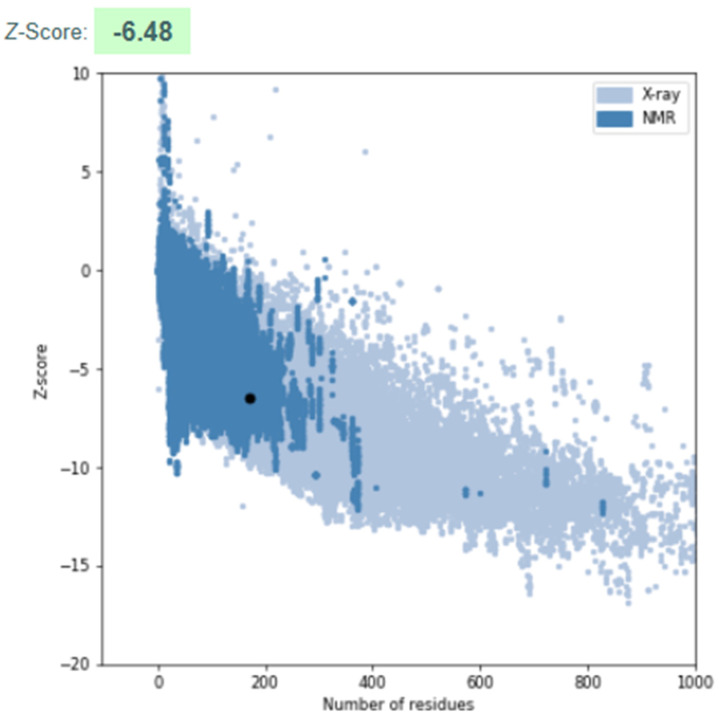
ProSA-Web validation of the refined 3D protein structure. The plot displays the Z-score (−6.48) against a reference dataset of experimentally determined structures. The Z-score confirms the model’s high quality, indicating its reliability for further functional and biochemical studies. Structure validation was performed using ProSA-Web.

The refined 3D structure was validated using ProSA-Web, which provided a Z-score of −6.48 (Figure 6). This Z-score indicates that the structure is within the expected range for high-quality protein models, confirming its reliability and accuracy. The Z-score value suggests that the model’s overall geometry and stereochemistry align with known protein structures, ensuring its suitability for further functional and biochemical studies.

### 3.4. B-Cell Epitope Prediction

The Bepipred Linear Epitope Prediction 2.0 algorithm was employed to identify potential B-cell epitopes within the PpiA protein sequence. Among the predicted epitopes, TGTGRGGPGYKFADEFHPELQF (residues 86–107) emerged as the most promising, with the highest prediction score of 0.760. This score indicates strong potential for immunogenicity. Positioned in a surface-exposed region of the protein, this epitope is accessible to immune cells. Its secondary structure features both helix and coil elements, which are associated with flexibility and accessibility, further enhancing its potential as a B-cell target (Table 1).

**Figure 7:**
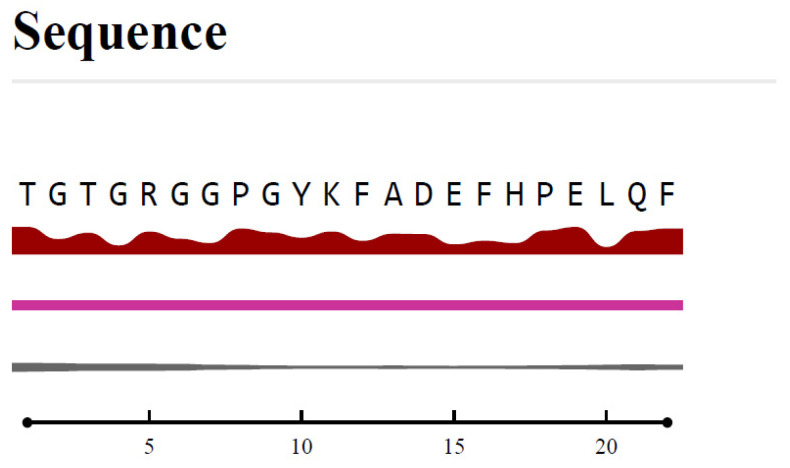
Epitope localization within the predicted structure of PpiA, highlighting its surface-exposed nature, which enhances accessibility to immune cells. The sequence analysis indicates that this epitope comprises both helix and coil regions, structural elements associated with flexibility and antigenic potential. The relative surface accessibility is represented, with red denoting exposed residues and blue indicating buried regions (thresholded at 25%). Epitope localization and accessibility analysis were carried out using BepiPred 2.0, IEDB, and PyMOL.

This epitope was located in a surface-exposed region of the protein, making it accessible to immune cells (Figure 7). Additionally, its secondary structure includes both helix and coil, which are typically associated with flexibility and accessibility, further enhancing its potential as a B-cell target.

Conservation analysis using ConSurf confirmed that this epitope is highly conserved across different strains of Mtb, making it relevant for a broad range of Mtb variants. Furthermore, the epitope was assessed for allergenicity and toxicity using AllergenOnline and AllerTOP, and it was found to be non-allergenic and non-toxic, ensuring its safety for therapeutic use. Given these favorable characteristics, TGTGRGGPGYKFADEFHPELQF is considered a strong candidate for further experimental validation in vaccine or therapeutic development targeting PpiA in Mtb.

The second most promising epitope, QGTKDYSTQNASGGPSGPFY (residues 47–66), also showed a high prediction score of 0.750. It is similarly located in a surface-exposed region, making it a good candidate for immune recognition. Conservation analysis using ConSurf confirmed that both epitopes are highly conserved across different strains of Mtb, increasing their relevance for targeting a broad range of Mtb variants.

In addition to its high VaxiJen score, epitope 86–107 was selected for further analysis due to its favorable surface accessibility, structural features (including helix and coil regions), high conservation across Mtb strains (as confirmed by ConSurf), and non-allergenic, non-toxic profile as predicted by AllergenOnline and AllerTOP. While epitope 47–66 also showed promising characteristics, including a high VaxiJen score and surface exposure, epitope 86–107 demonstrated a slightly superior overall immunogenic profile, making it a stronger candidate for downstream analysis.

### 3.5. MHC Class I and Class II Epitope Prediction

In addition to B-cell epitopes, the potential for T-cell-mediated immunity was explored using the IEDB MHC epitope prediction tools. MHC Class I epitopes, which are crucial for stimulating CD8+ T-cells, were predicted from the PpiA protein sequence. Among the predicted peptides, FADEFHPEL (residues 97–105) scored the highest with a prediction score of 0.865, suggesting strong binding affinity to the HLA-A*02:01 allele. This peptide also exhibited a low percentile rank (0.05%), indicating its high binding affinity and strong potential to activate cytotoxic T-cell responses (Table 2). Another notable MHC Class I epitope was LQFDKPYLL (residues 105–113), which also showed a high binding score of 0.862 (percentile rank: 0.05%).

MHC Class II epitopes, which are involved in CD4+ T-cell activation, were also predicted using the IEDB MHC-II prediction tool. Among these, DKPYLLAMANAGPGT (residues 108–122) demonstrated the highest prediction score of 0.9713 and a percentile rank of 0.08%, indicating its strong potential to bind to HLA-DRB1*01:01 and elicit a robust CD4+ T-cell response. Other high-scoring MHC II epitopes included FDKPYLLAMANAGPG (residues 107–121) with a score of 0.963 and QFDKPYLLAMANAGP (residues 106–120), scoring 0.8837, both of which also fall within a favorable percentile rank range for T-cell activation (Table 3).

### 3.6. Immunogenicity and Selection of Candidate Peptides

Based on the epitope prediction scores for both B-cell and T-cell responses, several peptides emerge as promising candidates for immunological purposes. Among the B-cell epitopes, the peptide TGTGRGGPGYKFADEFHPELQF (residues 86–107), with the highest VaxiJen score of 0.760, remains the top contender for eliciting an antibody-mediated immune response. This epitope is not only highly predicted to be immunogenic but also lies in a surface-exposed region, allowing it to be readily accessible to immune cells. Additionally, its secondary structure features both helix and coil elements, which are critical for flexibility and recognition, further enhancing its potential as a B-cell target.

For T-cell-mediated immunity, the peptide FADEFHPEL (residues 97–105) stands out for MHC Class I binding with a high score of 0.865, making it a strong candidate for inducing cytotoxic T-cell responses. This peptide also exhibits a very low percentile rank (0.05%), highlighting its strong potential for activating CD8+ T-cells.

For MHC Class II T-cell responses, DKPYLLAMANAGPGT (residues 108–122) is the leading candidate, scoring 0.9713, with a low percentile rank (0.08%), making it an excellent choice for stimulating CD4+ T-cells.

Given the favorable characteristics of these peptides, TGTGRGGPGYKFADEFHPELQF for B-cell activation, FADEFHPEL for CD8+ T-cells, and DKPYLLAMANAGPGT for CD4+ T-cells are considered the best candidates for further experimental validation in the development of vaccines or immunotherapies targeting PpiA in Mtb (Table 4). Additionally, combining these peptides into a multi-epitope cocktail could significantly enhance the overall immune response. By targeting both humoral and cellular immunity, such a cocktail could offer broader protection across different HLA alleles and Mtb strains.

## 4. Discussion

In this study, we analyzed the structural and immunological properties of the Mtb protein PpiA, highlighting its potential as a vaccine target. Our findings indicate that PpiA is a structurally stable, highly conserved protein with promising antigenic properties, making it an attractive candidate for further experimental validation.

**Structural Integrity and Functional Implications**

The structural analysis of PpiA showed a well-folded architecture with abundant alpha-helices and beta-sheets, ensuring stability. The tertiary structure prediction yielded a GMQE score of 0.93, indicating strong homology. Validation via QMEANDisCo and MolProbity confirmed reliability, with 93.49% of residues in favored Ramachandran regions, signifying minimal steric clashes and high geometric accuracy.

These structural features are significant as they suggest that PpiA may play an important role in the bacterial stress response and protein folding, functions commonly associated with peptidyl-prolyl isomerases. Given its high level of conservation across Mtb strains, it is possible that PpiA contributes to bacterial survival and virulence, making it a potentially valuable target not only for vaccines but also for drug development. Further studies are needed to elucidate PpiA’s precise biological function in host–pathogen interactions.

### 4.1. Immunogenic Potential and Epitope Accessibility

One of the key findings of this study was the identification of immunodominant epitopes within PpiA that may elicit strong immune responses. The predicted B-cell epitope TGTGRGGPGYKFADEFHPELQF (residues 86–107) was found in a surface-exposed region, enhancing its accessibility to immune cells. This positioning suggests that PpiA could naturally stimulate antibody production during Mtb infection, supporting its potential role in humoral immunity.

Moreover, MHC Class I and Class II epitopes were identified with strong binding affinities, suggesting that PpiA may also contribute to cell-mediated immunity. The FADEFHPEL epitope (97–105) showed high binding affinity to HLA-A*02:01, indicating that it could effectively stimulate CD8+ cytotoxic T-cell responses. Similarly, DKPYLLAMANAGPGT (108–122) exhibited strong binding to HLA-DRB1*01:01, suggesting potential activation of CD4+ helper T-cells. The ability of PpiA to induce both humoral and cellular immunity is a valuable characteristic, as an ideal TB vaccine should trigger both antibody-mediated and T-cell-mediated responses.

Similar in silico analyses of well-known Mtb antigens, such as ESAT-6, Ag85B, and CFP-10, have demonstrated their capacity to induce both B-cell and T-cell responses, supporting their role in TB vaccine development [12]. For example, studies have shown that ESAT-6 contains multiple surface-exposed T-cell epitopes with high binding affinity to various HLA alleles, while Ag85B has been predicted to harbor both conserved and immunogenic regions that activate CD4+ T-cell responses [34]. Compared to these antigens, PpiA displays comparable epitope accessibility and immunogenicity, particularly through its ability to induce potential CD8+ and CD4+ T-cell responses, further supporting its candidacy as a novel TB vaccine target.

### 4.2. Comparison with Existing Vaccine Strategies

The current BCG vaccine, developed over a century ago, remains the only licensed vaccine for TB. While BCG provides partial protection, especially against severe TB in children, its efficacy varies significantly among different populations and fails to provide long-term protection against pulmonary TB in adults. As a result, alternative vaccine strategies, including subunit vaccines, viral vector vaccines, and mRNA-based approaches, are under investigation.

Most TB subunit vaccine candidates, including M72/AS01E, H56:IC31, ID93+GLA-SE, and TB10.4, target well-known virulence factors such as ESAT-6, Ag85B, CFP-10, and TB10.4, all of which play crucial roles in Mtb invasion and immune evasion but present unique challenges [35,36,37,38]. M72/AS01E, despite showing 50% efficacy in a Phase IIb trial, faces challenges related to limited durability of immune protection, necessitating further validation in larger trials [39]. H56:IC31, a multi-antigen vaccine, aims to boost immunity against latent TB but struggles with suboptimal CD8+ T-cell activation, crucial for intracellular bacterial clearance [35]. ID93+GLA-SE targets a broader immune response; however, its protective efficacy remains uncertain due to the lack of established immune correlates for TB, absence of BCG-vaccinated or Mtb-exposed participants, and variability in immunogenicity across populations [40]. ESAT-6-based vaccines face challenges due to the antigen’s role in immune evasion. ESAT-6, in complex with CFP-10, inhibits IL-12 production, impairing the host’s ability to mount an effective immune response [41]. Additionally, ESAT-6 disrupts phagosomal membrane integrity, facilitating Mtb escape into the cytosol and further evading immune responses [42]. Similarly, Ag85B vaccines suffer from genetic variability across Mtb strains, leading to inconsistent immune recognition [35]. Lastly, TB10.4-based vaccines, despite strong immunogenicity, exhibit low induction of memory T-cell responses, limiting long-term protection [43]. Another challenge with current TB vaccine strategies is the limited induction of strong CD8+ T-cell responses, which are critical for eliminating intracellular Mtb [44]. Many TB vaccine candidates focus on CD4+ T-cell activation; however, CD8+ T-cells possess unique effector mechanisms, which are essential for controlling Mtb infection. Therefore, vaccine strategies that do not adequately stimulate CD8+ T-cell responses may be less effective in providing comprehensive protection against TB. Additionally, antigen accessibility is a major consideration in vaccine design. Several current TB vaccine targets are buried within the bacterial cell wall, reducing their direct exposure to the host immune system. The complex lipid-rich composition of the Mtb cell wall can impede the presentation of antigens to the host’s immune cells, thereby affecting the efficacy of vaccine-induced immune responses [45,46]. Therefore, selecting antigens that are readily accessible and capable of eliciting robust immune responses is crucial for the development of effective TB vaccines. Given the limitations of current TB vaccines, PpiA offers key advantages. It is highly conserved across Mtb strains, reducing the risk of immune escape. Unlike variable antigens like Ag85B, PpiA’s stability enhances its reliability as a vaccine component. PpiA’s strong immunogenicity enables robust CD4+ and CD8+ T-cell activation, crucial for TB defense. Its ability to bind multiple HLA alleles supports broad population coverage, addressing a major challenge in TB vaccine design. Additionally, PpiA’s surface-exposed epitopes enhance both antibody- and T-cell-mediated immunity, making it more accessible to immune surveillance and improving its potential as a protective antigen. Furthermore, given that TB is a complex intracellular infection requiring both innate and adaptive immune responses, a multi-antigen approach may offer the best protection [47,48,49,50]. PpiA-based vaccines could be combined with existing antigens, such as ESAT-6, Ag85B, or CFP-10, to enhance immunogenicity and address the shortcomings of current candidates. Additionally, PpiA could serve as an adjuvant antigen in novel vaccine platforms, including mRNA-based vaccines or viral vector vaccines, to boost CD8+ T-cell responses. As seen with COVID-19 vaccines, mRNA platforms offer the advantages of rapid antigen production and flexible design.

Given the intracellular nature of Mtb, mRNA vaccine platforms offer a promising avenue for antigen delivery and immune activation. While our findings suggest that PpiA possesses structural and immunological features compatible with such platforms, further experimental validation is essential to confirm its suitability for RNA-based vaccine development.

### 4.3. Future Directions and Challenges

While our findings support the immunogenic potential of PpiA, further validation is essential. In vitro studies should include ELISA and T-cell proliferation assays to confirm whether the predicted epitopes elicit immune responses in human immune cells, along with cytotoxic T lymphocyte (CTL) assays to assess CD8+ T-cell-mediated killing of infected cells. In vivo studies using mouse and non-human primate models are needed to evaluate the protective efficacy of PpiA-based vaccines, particularly through challenge studies with virulent Mtb strains to determine bacterial load reduction and survival benefits. Additionally, structural and functional characterization of PpiA should explore its role in Mtb pathogenesis, host interactions, and potential enzymatic activity. Finally, a multi-epitope vaccine approach incorporating PpiA-derived epitopes with established antigens like ESAT-6, Ag85B, or CFP-10 could enhance immune response breadth, providing stronger protection against TB.

While this study primarily focused on human TB, it is important to acknowledge the growing concern of tuberculosis in farm animals, which presents both public health and veterinary challenges. Future research could benefit from exploring the immunological aspects of *PpiA* across species, as animal models could provide valuable insights into the pathogenesis and treatment of tuberculosis

### 4.4. Conclusions

Our study highlights PpiA as a promising TB vaccine target, owing to its structural stability, high conservation, and strong immunogenic potential. The identification of surface-exposed B-cell and T-cell epitopes suggests that PpiA could contribute to both humoral and cellular immunity, addressing key limitations of current TB vaccines. Given its potential to induce strong CD8+ T-cell responses, PpiA may complement existing vaccine strategies and improve protection against TB.

While these findings provide a strong rationale for further investigation, experimental validation is essential to confirm the immunogenicity and protective efficacy of PpiA-based vaccine candidates. If successful, PpiA could represent a novel addition to the TB vaccine pipeline, offering a more universal and broadly applicable antigen to combat TB on a global scale.

## Figures and Tables

**Table 1:** Predicted B-cell epitopes in PpiA protein and their VaxiJen scores.

No.	Start	End	Peptide	Length	VaxiJen Score
1	86	107	TGTGRGGPGYKFADEFHPELQF	22	0.760
2	47	66	QGTKDYSTQNASGGPSGPFY	20	0.750
3	164	169	TDGNDR	6	0.535
4	5	11	DSVTNSP	7	0.475
5	135	137	PHL	3	0.392

**Table 2:** MHC Class I epitope prediction.

No.	Start	End	Peptide	Length	Core Sequence	Score	Percentile Rank
1	97	105	FADEFHPEL	9	FADEFHPEL	0.865	0.05
2	105	113	LQFDKPYLL	9	LQFDKPYLL	0.862	0.05
3	29	39	ALFGNHAPKTV	11	ALFGAPKTV	0.535	0.24
4	147	155	VIDAESQRV	9	VIDAESQRV	0.377035	0.4

**Table 3:** MHC Class II epitope prediction.

No.	Start	End	Peptide Sequence	Length	Core Sequence	Score	Percentile Rank
1	108	122	DKPYLLAMANAGPGT	15	YLLAMANAG	0.9713	0.08
2	107	121	FDKPYLLAMANAGPG	15	YLLAMANAG	0.9630	0.12
3	106	120	QFDKPYLLAMANAGP	15	YLLAMANAG	0.8837	0.55
4	109	123	KPYLLAMANAGPGTN	15	YLLAMANAG	0.8551	0.64

**Table 4:** Immunogenicity and peptide characteristics.

Peptide	Residues	Type of Immunity	VaxiJen Score/Binding Score	Percentile Rank	Target Immune Cell	Additional Characteristics
**TGTGRGGPGYKFADEFHPELQF**	86–107	B-cell immunity	0.760	N/A	B-cells	Surface-exposed, flexible secondary structure (helix + coil), high immunogenicity potential
**FADEFHPEL**	97–105	Cytotoxic T-cell (CD8+)	0.865	0.05%	CD8+ T-cells	Strong MHC Class I binding, low percentile rank, high potential for inducing cytotoxic T-cell response
**DKPYLLAMANAGPGT**	108–122	Helper T-cell (CD4+)	0.9713	0.08%	CD4+ T-cells	Strong MHC Class II binding, low percentile rank, excellent candidate for CD4+ T-cell activation

## Data Availability

Data available in the figures.

## Abbreviations

The following abbreviations are used in this manuscript:

PpiA	Peptidyl-prolyl isomerase A
Mtb	*Mycobacterium tuberculosis*
TB	Tuberculosis
MHC	Major histocompatibility complex

## References

[1] World Health Organization (2024). Global Tuberculosis Report.

[2] Dheda K., Mirzayev F., Cirillo D.M., Udwadia Z., Dooley K.E., Chang K.-C., Omar S.V., Reuter A., Perumal T., Horsburgh C.R. (2024). Multidrug-resistant tuberculosis. Nat. Rev. Dis. Primers.

[3] Lv H., Zhang X., Zhang X., Bai J., You S., Li X., Li S., Wang Y., Zhang W., Xu Y. (2024). Global prevalence and burden of multidrug-resistant tuberculosis from 1990 to 2019. BMC Infect. Dis..

[4] Nasiri M.J., Zangiabadian M., Arabpour E., Amini S., Khalili F., Centis R., D’Ambrosio L., Denholm J.T., Schaaf H.S., van den Boom M. (2022). Delamanid-containing regimens and multidrug-resistant tuberculosis: A systematic review and meta-analysis. Int. J. Infect. Dis..

[5] Nasiri M.J., Lutfy K., Venketaraman V. (2024). Challenges of Multidrug-Resistant Tuberculosis Meningitis: Current Treatments and the Role of Glutathione as an Adjunct Therapy. Vaccines.

[6] Seeberg J. (2023). An epidemic of drug resistance: Tuberculosis in the twenty-first century. Pathogens.

[7] Nasiri M.J., Venketaraman V. (2025). Advances in Host–Pathogen Interactions in Tuberculosis: Emerging Strategies for Therapeutic Intervention. Int. J. Mol. Sci..

[8] Sharma A.K., Mal S., Sahu S.K., Bagchi S., Majumder D., Chakravorty D., Saha S., Kundu M., Basu J. (2025). Mycobacterial peptidyl prolyl isomerase A activates STING-TBK1-IRF3 signaling to promote IFNβ release in macrophages. FEBS J..

[9] Dubey N., Khan M.Z., Kumar S., Sharma A., Das L., Bhaduri A., Singh Y., Nandicoori V.K. (2021). Mycobacterium tuberculosis peptidyl prolyl isomerase a interacts with host integrin receptor to exacerbate disease progression. J. Infect. Dis..

[10] Pandey S., Tripathi D., Khubaib M., Kumar A., Sheikh J.A., Sumanlatha G., Ehtesham N.Z., Hasnain S.E. (2017). Mycobacterium tuberculosis peptidyl-prolyl isomerases are immunogenic, alter cytokine profile and aid in intracellular survival. Front. Cell. Infect. Microbiol..

[11] Pandey S., Sharma A., Tripathi D., Kumar A., Khubaib M., Bhuwan M., Chaudhuri T.K., Hasnain S.E., Ehtesham N.Z. (2016). Mycobacterium tuberculosis peptidyl-prolyl isomerases also exhibit chaperone like activity in-vitro and in-vivo. PLoS ONE.

[12] Nugraha M.F., Changestu D.A., Ramadhan R., Salsabila T., Nurizati A., Pratiwi S.E., Ysrafil Y. (2024). Novel prophylactic and therapeutic multi-epitope vaccine based on Ag85A, Ag85B, ESAT-6, and CFP-10 of Mycobacterium tuberculosis using an immunoinformatics approach. Osong Public Health Res. Perspect..

[13] Zhu B., Dockrell H.M., Ottenhoff T.H.M., Evans T.G., Zhang Y. (2018). Tuberculosis vaccines: Opportunities and challenges. Respirology.

[14] Ouyang J., Guo S., Hu Z., Cao T., Mou J., Gu X., Huang C., Liu J. (2025). Recombinant protein Ag85B-Rv2660c-MPT70 promotes quality of BCG-induced immune response against Mycobacterium tuberculosis H37Ra. Front. Immunol..

[15] Phogat S., Yadav J., Chaudhary D., Jaiwal R., Jaiwal P.K. (2025). Synthesis of an Adjuvant-Free Single Polypeptide-Based Tuberculosis Subunit Vaccine that Elicits In Vivo Immunogenicity in Rats. Molecular Biotechnology.

[16] Cardona P.J. (2018). Pathogenesis of tuberculosis and other mycobacteriosis. Enferm. Infecc. Microbiol. Clin..

[17] Chirani A.S., Majidzadeh R., Pouriran R., Heidary M., Nasiri M.J., Gholami M., Goudarzi M., Omrani V.F. (2018). The effect of in silico targeting Pseudomonas aeruginosa patatin-like protein D, for immunogenic administration. Comput. Biol. Chem..

[18] Rahman M.S., Rahman M.K., Saha S., Kaykobad M., Rahman M.S. (2019). Antigenic: An improved prediction model of protective antigens. Artif. Intell. Med..

[19] Safari N., Ardakan A.K., Hamedi E., Kalantarzadeh F., Kaveh P., Rahmanian P., Ghiabi S., Hosseini S.A., Siamian D., Gorgipour M. (2024). An in silico approach to decipher immunogenic epitopes in Toxoplasma gondii GRA1 and GRA3. Inform. Med. Unlocked.

[20] Azimi R., Ozgul M., Kenney M.C., Kuppermann B.D. (2024). Bioinformatic analysis of small humanin like peptides using AlfaFold-2 and Expasy ProtParam. Investig. Ophthalmol. Vis. Sci..

[21] Gasteiger E., Hoogland C., Gattiker A., Duvaud S.e., Wilkins M.R., Appel R.D., Bairoch A. (2005). Protein Identification and Analysis Tools on the ExPASy Server.

[22] Morita R., Shigeta Y., Harada R. (2021). Comprehensive predictions of secondary structures for comparative analysis in different species. J. Struct. Biol..

[23] Guex N., Peitsch M.C., Schwede T. (2009). Automated comparative protein structure modeling with SWISS-MODEL and Swiss-PdbViewer: A historical perspective. Electrophoresis.

[24] Biasini M., Bienert S., Waterhouse A., Arnold K., Studer G., Schmidt T., Kiefer F., Cassarino T.G., Bertoni M., Bordoli L. (2014). SWISS-MODEL: Modelling protein tertiary and quaternary structure using evolutionary information. Nucleic Acids Res..

[25] Waterhouse A., Bertoni M., Bienert S., Studer G., Tauriello G., Gumienny R., Heer F.T., de Beer T.A.P., Rempfer C., Bordoli L. (2018). SWISS-MODEL: Homology modelling of protein structures and complexes. Nucleic Acids Res..

[26] Carrascoza F., Zaric S., Silaghi-Dumitrescu R. (2014). Computational study of protein secondary structure elements: Ramachandran plots revisited. J. Mol. Graph. Model..

[27] Chen V.B., Arendall W.B., Headd J.J., Keedy D.A., Immormino R.M., Kapral G.J., Murray L.W., Richardson J.S., Richardson D.C. (2010). MolProbity: All-atom structure validation for macromolecular crystallography. Acta Crystallogr. Sect. D Biol. Crystallogr..

[28] Williams C.J., Headd J.J., Moriarty N.W., Prisant M.G., Videau L.L., Deis L.N., Verma V., Keedy D.A., Hintze B.J., Chen V.B. (2018). MolProbity: More and better reference data for improved all-atom structure validation. Protein Sci..

[29] Wiederstein M., Sippl M.J. (2007). ProSA-web: Interactive web service for the recognition of errors in three-dimensional structures of proteins. Nucleic Acids Res..

[30] Kim Y., Ponomarenko J., Zhu Z., Tamang D., Wang P., Greenbaum J., Lundegaard C., Sette A., Lund O., Bourne P.E. (2012). Immune epitope database analysis resource. Nucleic Acids Res..

[31] Vita R., Zarebski L., Greenbaum J.A., Emami H., Hoof I., Salimi N., Damle R., Sette A., Peters B. (2010). The immune epitope database 2.0. Nucleic Acids Res..

[32] Potocnakova L., Bhide M., Pulzova L.B. (2016). An introduction to B-cell epitope mapping and in silico epitope prediction. J. Immunol. Res..

[33] Vita R., Mahajan S., Overton J.A., Dhanda S.K., Martini S., Cantrell J.R., Wheeler D.K., Sette A., Peters B. (2019). The immune epitope database (IEDB): 2018 update. Nucleic Acids Res..

[34] Yun J.S., Kim A.R., Kim S.M., Shin E., Ha S.J., Kim D., Jeong H.S. (2024). In silico analysis for the development of multi-epitope vaccines against Mycobacterium tuberculosis. Front. Immunol..

[35] Zhang Y., Xu J.-c., Hu Z.-d., Fan X.-y. (2023). Advances in protein subunit vaccines against tuberculosis. Front. Immunol..

[36] Kim H., Choi H.-G., Shin S.J. (2023). Bridging the gaps to overcome major hurdles in the development of next-generation tuberculosis vaccines. Front. Immunol..

[37] Romano M., Squeglia F., Kramarska E., Barra G., Choi H.-G., Kim H.-J., Ruggiero A., Berisio R. (2023). A structural view at vaccine development against M. tuberculosis. Cells.

[38] Jurczak M., Druszczynska M. (2025). Beyond Tuberculosis: The Surprising Immunological Benefits of the Bacillus Calmette–Guérin (BCG) Vaccine in Infectious, Auto-Immune, and Inflammatory Diseases. Pathogens.

[39] WHO Global Tuberculosis Programme; New TB Vaccine Research. https://www.who.int/teams/global-tuberculosis-programme/research-innovation/vaccines?utm_source=chatgpt.com.

[40] Sagawa Z.K., Goman C., Frevol A., Blazevic A., Tennant J., Fisher B., Day T., Jackson S., Lemiale F., Toussaint L. (2023). Safety and immunogenicity of a thermostable ID93+ GLA-SE tuberculosis vaccine candidate in healthy adults. Nat. Commun..

[41] Pal R., Bisht M.K., Mukhopadhyay S. (2022). Secretory proteins of Mycobacterium tuberculosis and their roles in modulation of host immune responses: Focus on therapeutic targets. FEBS J..

[42] Passos B.B., Araújo-Pereira M., Vinhaes C.L., Amaral E.P., Andrade B.B. (2024). The role of ESAT-6 in tuberculosis immunopathology. Front. Immunol..

[43] Shi S., Yu L., Sun D., Liu J., Hickey A.J. (2010). Rational design of multiple TB antigens TB10. 4 and TB10. 4-Ag85B as subunit vaccine candidates against Mycobacterium tuberculosis. Pharm. Res..

[44] Kudryavtsev I., Zinchenko Y., Serebriakova M., Akisheva T., Rubinstein A., Savchenko A., Borisov A., Belenjuk V., Malkova A., Yablonskiy P. (2023). A key role of cd8+ t cells in controlling of tuberculosis infection. Diagnostics.

[45] Liu H., Gui X., Chen S., Fu W., Li X., Xiao T., Hou J., Jiang T. (2022). Structural Variability of Lipoarabinomannan Modulates Innate Immune Responses within Infected Alveolar Epithelial Cells. Cells.

[46] Vergne I., Chua J., Singh S.B., Deretic V. (2004). Cell biology of Mycobacterium tuberculosis phagosome. Annu. Rev. Cell Dev. Biol..

[47] Chugh S., Bahal R.K., Dhiman R., Singh R. (2024). Antigen identification strategies and preclinical evaluation models for advancing tuberculosis vaccine development. NPJ Vaccines.

[48] Yun J.-S., Shin E., Lee Y.-R., Lee J.-A., Lee H., Kim J.-S., Shin S.J., Ha S.-J., Lee S.-W., Kim D. (2025). Immunogenicity and protective efficacy of a multi-antigenic adenovirus-based vaccine candidate against Mycobacterium tuberculosis. Front. Microbiol..

[49] Pillay K., Chiliza T.E., Senzani S., Pillay B., Pillay M. (2024). In silico design of Mycobacterium tuberculosis multi-epitope adhesin protein vaccines. Heliyon.

[50] An Y., Ni R., Zhuang L., Yang L., Ye Z., Li L., Parkkila S., Aspatwar A., Gong W. (2025). Tuberculosis vaccines and therapeutic drug: Challenges and future directions. Mol. Biomed..

